# Clonal integration promotes the growth of *Phragmites australis* populations in saline wetlands of the Yellow River Delta

**DOI:** 10.3389/fpls.2023.1162923

**Published:** 2023-06-02

**Authors:** Bo Guan, Junbao Yu, Mengdi Wu, Xiaoling Liu, Xuehong Wang, Jisong Yang, Di Zhou, Xiaolong Zhang

**Affiliations:** ^1^The Institute for Advanced Study of Coastal Ecology, Ludong University, Yantai, China; ^2^School of Environmental and Material Engineering, Yantai University, Yantai, China; ^3^Key Laboratory of Coastal Environmental Processes and Ecological Remediation, Yantai Institute of Coastal Zone Research (YIC), Chinese Academy of Sciences (CAS), Shandong Key Laboratory of Coastal Environmental Processes, YICCAS, Yantai, Shandong, China

**Keywords:** *Phragmites australis*, salt heterogeneity, clonal integration, ecological adaptation, ion content

## Abstract

Estuarine wetlands are highly heterogeneous due to strong interactions between freshwater input and seawater intrusion. However, little is known about how clonal plant populations adapt to heterogeneous salinity in soil environments. In the present study, the effects of clonal integration on *Phragmites australis* populations under salinity heterogeneity were studied using field experiments with 10 treatments in the Yellow River Delta. Clonal integration significantly increased plant height, aboveground biomass, underground biomass, root–shoot ratio, intercellular CO_2_ concentration, net photosynthetic rate, stomatal conductance, transpiration rate, and stem Na^+^ content under homogeneous treatment. Under the heterogeneous salt treatment, clonal integration significantly affected total aboveground and underground biomass, photosynthetic traits, and stem Na^+^ content under different salt gradients. The increase in salt concentration inhibited the physiological activity and growth of *P. australis* to varying degrees. Compared with the heterogeneous saline environment, clonal integration was more beneficial to *P. australis* populations in the homogeneous saline habitat. The results of the present study suggest that *P. australis* prefers homogeneous saline habitats; however, plants can adapt to heterogeneous salinity conditions *via* clonal integration.

## 1 Introduction

Soil heterogeneity is prevalent in natural habitats and exists at a fine scale (Hutchings and Wijesinghe, 1997; Alpert, 1999; Dong et al., 2015; Keser et al., 2015); soil heterogeneity can also enhance plant growth or reproduction through plasticity in root growth or physiological changes, and facilitate efficient use of unevenly distributed resources, based on greenhouse or garden studies (Yu et al., 2001; Shen et al., 2020). For example, Yu et al. (2020) observed that nutrient heterogeneity significantly enhanced the morphological traits (plant height and number of ramets, spacers, and length) and biomass accumulation of *Typha orientalis* Presal (Yu et al., 2020). Wang et al. (2016) also observed a similar phenomenon: when compared with those in the homogeneous environment, *Iris japonica* biomass, ramet number and rhizome length increased in different degrees within large patch heterogeneous areas (Wang et al., 2016). However, we observed contrasting results in our previous study, with *Phragmites australis* preferring homogeneous nutrient conditions in a saline micro-environment, based on a split-root experiment (Guan et al., 2021). So far, the responses of different plants to environmental heterogeneity have mostly been conducted based on pot experiments at the individual scale (Yu et al., 2001; Liu et al., 2017; Shen et al., 2020). However, the adaptation of plants to environmental heterogeneity at the population scale has rarely been studied.

In estuarine wetlands, climate change, human activity, storm surge inundation, and the interaction of terrestrial and marine environments all jointly introduce both resource and stressor heterogeneity (Tho et al., 2008; Wang et al., 2009; Yu et al., 2014). The different degrees of spatial heterogeneity in environmental factors present a major challenge for plant growth and survival (Yu et al., 2016; Shen et al., 2020; Guan et al., 2021). In estuarine wetlands, soil heterogeneity and high soil salinity limit the growth of most plants and vegetation is mainly composed of salt-tolerant species (Koevoets et al., 2016). Clonal plants account for a substantial proportion of such species, considering their unique adaptation strategies to heterogeneous wetland environments, through the production of genetically identical individual subunits *via* clonal growth (Liang et al., 2020). Clonal integration, also referred to as physiological integration, is a unique feature *via* which clonal plants adapt to adverse environments by sharing resources among connected ramets (Wang et al., 2017). Numerous studies have demonstrated that clonal plants can adjust the biomass of the resource-acquiring organs to high nutrition areas in nutrient heterogeneous environments (Alpert, 1999; Wang et al., 2008; Keser et al., 2015; Shen et al., 2020). At present, research on the adaptation of clonal plants to heterogeneous environments has mostly focused on nutrients (Liang et al., 2020), water (You et al., 2016), and light (Chen et al., 2019), with relatively little research focusing on the heterogeneity of coastal wetland vegetation under stress factors such as salinity.

*P. australis* is a typical salt-tolerant clonal plant with high soil salinity tolerance and the capacity to survive extreme salinity environments (Li et al., 2009; Eller et al., 2017; Liu et al., 2021). In heterogeneous habitats, *P. australis* can adjust the sizes of resource-acquisition organs (such as the length and diameter of roots and rhizomes), the distribution and number of ramets, and other morphological indicators (such as shoot diameter, plant height, and leaf area) to cope with resource heterogeneity (Zhang et al., 2018; Chen et al., 2019; Yu et al., 2020; Wang et al., 2021). However, whether *P. australis* could colonize unfavorable habitats through clonal integration when compared to non-clonal individuals is still unclear. Moreover, studies on the effect of salinity heterogeneity on *P. australis* under different salinity levels could enhance our understanding of the adaptive mechanisms of clonal plants in heterogeneous saline habitats.

To investigate the effects of clonal integration on plant communities under different stress conditions, which could further provide a theoretical basis for the ecological restoration of coastal wetlands, we conducted a study on *P. australis* in different salinity environments in a typical estuarine wetland of the Yellow River Delta. Field control experiments were conducted on *P. australis* and the effects of clonal integration of *P. australis* populations in different homogeneous and heterogeneous salinity environments were tested. Specifically, the following two questions were addressed: (1) Does severance decrease *P. australis* growth and physiological traits in saline homogeneous and heterogeneous habitats? And (2) if so, do the effects increase with an increase in salt stress?

## 2 Materials and methods

### 2.1 Experimental design

**Figure 1:**
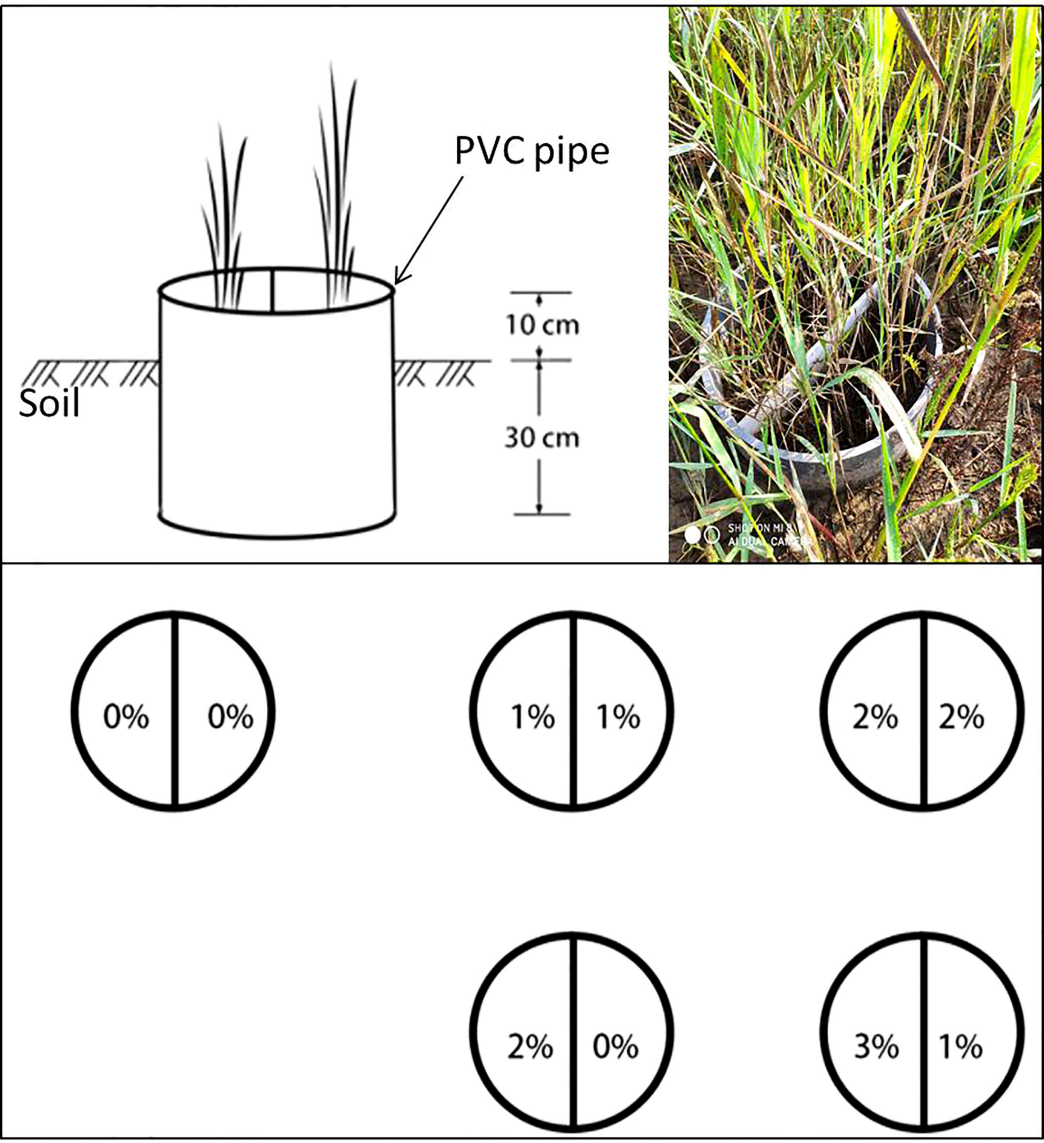
Schematic of the experimental design.

The study was conducted at the Yellow River Delta Ecology Research Station of Coastal Wetlands, Shandong, China (37° 45′ 50″ N, 118° 59′ 24″ E), which is located in the Nature Reserve of the Yellow River Delta. *P. australis* stands with uniform patches were selected as the experimental blocks. Forty circular plots (PVC pipes), with 40 cm diameter and 40 cm height, were randomly located within the blocks, with an at least 1-m interval between PVC pipes to minimize interference between treatments. To ensure that all the plots (PVC pipes) were at the same starting level and avoid interference by plants outside the plots, a narrow trench 30 cm deep was dug around the perimeter of each plot and the PVC pipes were inserted into the trenches, so that all the pipes extended 30 cm below and 10 cm above the soil surface, filling the trench and enclosing the plots (**Figure 1**). Before the experiment started, the aboveground part of the *P. australis* community was cut to ensure uniform growth, and about 15 cm of shoot was retained.

The experiment consisted of three factors: severed treatment (rhizomes either severed in the middle of the plot to prevent clonal integration or left connected), salt level, and salt homogeneity/heterogeneous treatment, including ten treatments with four replicates in a completely randomized design. For the severed treatment, a PVC plate (40 cm wide, 40 cm high) was inserted in the PVC pot and sealed with glass glue to prevent mixing of the salt solutions. For the connected treatment, a PVC plate (40 cm wide, 15cm high) with 5 cm below (taking care not to sever rhizomes) and 10 cm above the soil surface was inserted (**Figure 1**). In three homogeneous salt treatments, both halves of the pot were exposed to the same NaCl concentrations (0%, 1%, or 2%, marked with 0%/0%, 1%/1%, and 2%/2%, respectively), and in two heterogeneous salt treatments, one side was exposed to high NaCl concentration (2% or 3%), and the other to low NaCl concentration (0% or 1%), which were labeled as 2%/0% and 3%/1%, respectively.

The plants started to be watered on 1st June 2020 with different concentrations of NaCl solution (two halves received the same amount of solution) after 10 days of recovery. About a week later, the soil salt concentrations reached the desired treatment levels. To maintain the salt concentrations, water lost to evaporation loss was made up every three or four days, and to avoid significant changes in salt concentrations, a portable electrical conductivity meter was used to monitor the salt concentration every week and the soil salt concentration was adjusted with NaCl solution.

### 2.2 Data collection

The experiment lasted four months and measurement of photosynthetic traits was performed in sunny weather at 8:00-11:30 and 14:00-17:00 on 15th September 2020. P*. australis* leaves with similar growth were selected in each half. The net photosynthetic rate (*Pn*), intercellular CO_2_ concentration (*Ci*), stomatal conductance (*Gs*), and transpiration rate (*Tr*) of leaves under saturated light were measured using the TARGAS-1 portable photosynthetic apparatus (PP Systems, Amesbury, MA, USA). Plant height, number of ramets, number of leaves, leaf width and length, stem diameter, and internode length in each half were measured. The third or fourth fully expanded leaves from the top were selected for the leaf length and width measurements. After the experiments, the *P. australis* plants were divided into aboveground and underground parts. In the case of aboveground parts, the leaves and stems (naked stems and leaf sheaths included) were collected separately, and the rhizomes and adventitious roots in each half pot with a depth of 30 cm (the main root layer of *P. australis*) were collected as underground parts. The plant samples were oven-dried at 105 °C for 15 min, then dried at 60 °C to constant weight, and the dry weight was recorded. The concentrations of Na^+^ and K^+^ in *P. australis* stem and leaf were measured using an atomic absorption spectrophotometer (AA = 6800, Shimadzu, Japan). Proline content in leaves was determined using a 722 spectrophotometer (Shanghai Precision & Scientific Instrument Co., Ltd., Shanghai, China) at 520 nm.

### 2.3 Statistical analysis

Two-way analysis of variance was used to test the effects of clonal integration (severance vs. no severance) and salinity level (0%/0%, 1%/1%, and 2%/2%) in a homogeneous salt environment, the effects of clonal integration (severance vs. no severance) and salt level (2%/0% and 3%/1%) in a heterogeneous salt environment, and the effects of salt heterogeneity and salt level (1%/1%, 2%/2%, 2%/0%, and 3%/1%) on the aboveground and underground biomass, plant growth (plant height, number of leaves, stem diameter, root-shoot ratio), and physiological traits (photosynthesis, Na^+^ and K^+^ content in leaves and stems, and leaf proline content) of *P. australis*. Differences in plant growth and physiological traits between severed and intact treatments of the same salinity level were assessed using t-tests. Analyses were performed with IBM SPSS Statistics 20 (IBM Corp., Armonk, NY, USA) at a 0.05 level of significance.

## 3 Results

### 3.1 Plant growth

In general, plant height, leaf length, and stem diameter decreased significantly with increasing salt stress (*p* < 0.05, **Table 1**). Under homogeneous conditions, the severed treatment significantly decreased plant height in the salt-free treatment; however, no significant differences were observed among other plant growth traits, such as stem diameter, leaf length, leaf width, and internode length (*p* > 0.05).

**Table 1:** Two-way ANOVAs for the effects of the salt and severed treatments on plant growth and physiological traits of *Phragmites australis* with homogeneous salt treatments (0%/0%, 1%/1%, and 2%/2%).

Traits	Salt treatment	Severed treatment	Salt × Severed
Relative density growth rate	2.149	2.808	0.291
Plant height	13.007**	0.897**	0.208
Leaf length	5.402*	1.476	1.647
Leaf width	0.533	0.156	2.117
Number of leaves	0.125	3.125	0.875
Internode length	5.390*	1.105	1.977
Stem diameter	5.145*	4.772	0.001
Total underground biomass	45.270***	30.086***	11.477*
Total aboveground biomass	87.917***	6.644*	0.239
Root-shoot ratio	6.430*	12.881**	3.265
*Pn*	13.630**	13.638**	4.143*
*Ci*	11.785**	2.447	2.656
*Gs*	13.196**	5.536*	1.592
*Tr*	7.969**	50.013***	8.307**
Na-stem	59.385***	15.125**	0.307
Na-leaf	24.976***	0.435	0.024
K-stem	3.155	2.457	0.333
K-leaf	0.408	0.382	0.704
Proline	10.150**	0.521	2.096

*P<0.05, **P<0.01, ***P<0.001.

Bold value means the trait was significantly affected by the treatment.

**Figure 2:**
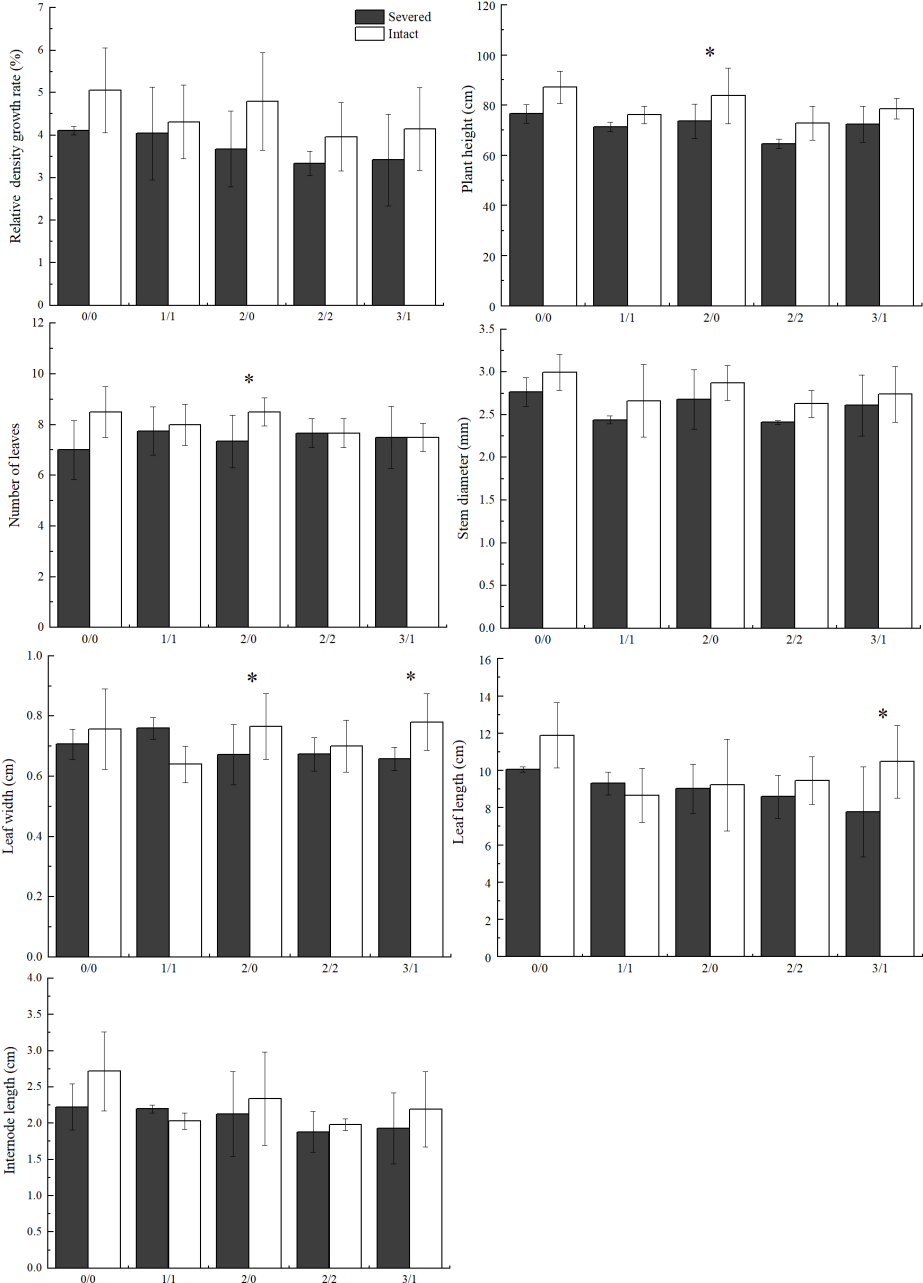
Effects of salt and severance on *Phragmites australis* growth under homogenous and heterogenenous salt treatments (mean ± SD). * represent significant differences between severed (■) and intact (□) treatments under the same salinity level at 0.05 level.

In the heterogeneous salt environment, the salt and severed treatment affected some growth traits of *P. australis* significantly (**Figure 2**). With an increase in salt stress, plant height decreased significantly (*p* < 0.05). Severed treatment significantly decreased plant height in the 2%/0% treatment (*p* < 0.05). With an increase in salt stress (3%/1% treatment), leaf length in *P. australis* of the intact group was significantly higher than that in the severed group (*p* < 0.05, **Figure 2**). However, the interaction of the salt and severed treatment did not significantly influence plant growth traits (*p* > 0.05, **Figure 2**, **Table 2**).

**Table 2:** Two-way ANOVAs for the effects of the salt and severed treatments on plant growth and physiological traits of *Phragmites australis* with heterogeneous salt treatments (2%/0% and 3%/1%).

Traits	Salt treatment	Severed treatment	Salt × Severed
Relative density growth rate	0.070	0.862	0.862
Plant height	8.630**	0.590	0.862
Leaf length	0.062	1.389	1.689
Leaf width	0.025	7.351*	0.314
Number of leaves	1.316	2.579	2.579
Internode length	0.724	1.345	0.016
Stem diameter	0.618	1.556	0.063
Total underground biomass	0.003	16.969***	0.004
Total aboveground biomass	2.587	14.132***	0.455
Root-shoot ratio	0.103	2.955	0.293
*Pn*	6.722*	15.375***	0.441
*Ci*	17.341***	2.753	0.041
*Gs*	1.116	1.695	0.092
*Tr*	11.266**	16.129***	0.169
Na-stem	5.186*	36.398***	3.067
Na-leaf	0.502	10.386**	0.561
K-stem	0.278	0.242	0.381
K-leaf	1.294	1.814	3.212
Proline	6.606*	3.506	0.000

*P<0.05, **P<0.01, ***P<0.001.

Bold value means the trait was significantly affected by the treatment.

### 3.2 Biomass allocation

**Figure 3:**
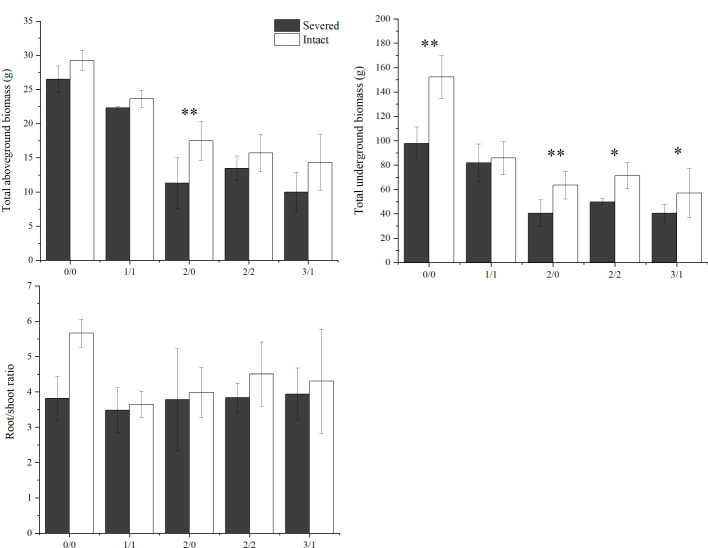
Effects of salt and severance on total aboveground and underground biomass of *Phragmites australis* and the root-shoot ratio under homogenous and heterogenenous salt treatments (mean ± SD). * and ** represent significant differences between severed (■) and intact (□) treatments under the same salinity level at 0.05 and 0.01 level.

The salt and severed treatment significantly decreased the total aboveground biomass, total underground biomass, and root-shoot ratio of *P. australis* (*p* < 0.05, **Figure 3**, **Table 1**). Under homogeneous conditions, the severed treatment significantly decreased the total underground biomass in the salt-free treatment (*p* < 0.05, **Figure 3**, **Table 1**); however, the aboveground biomass did not exhibit significant difference between the severed and the intact treatments.

Under heterogeneous salt conditions, the severed treatment significantly decreased the underground and aboveground biomass in both the 2%/0% and 3%/1% treatments (*p* < 0.05, **Table 2**). Moreover, the biomass (aboveground and underground) in the 1%/1% homogeneous salt treatment was significantly higher than that in the 2%/0% heterogeneous salt treatment (*p* < 0.05), and the aboveground biomass and the root-shoot ratio were affected significantly by the interaction of salt concentration and salt heterogeneity (**Table 3**).

**Table 3:** Two-way ANOVAs for the effects of salt concentration (SC) and salt heterogeneity (SH) on plant growth and physiological traits of *Phragmites australis* with different salt treatments (1%/1%, 2%/2%, 2%/0%, and 3%/1%).

Traits	Salt concentration	Salt heterogeneity	SC × SH
Relative density growth rate	0.984	0.444	0.093
Plant height	1.248	3.014	0.056
Leaf length	2.197	0.267	0.361
Leaf width	0.068	2.600	0.003
Number of leaves	4.392	0.275	1.098
Internode length	0.159	1.105	0.040
Stem diameter	0.348	1.232	0.11
Total underground biomass	3.410	12.366**	0.161
Total aboveground biomass	12.175**	24.856***	6.707*
Root-shoot ratio	0.795	1.527	9.666**
*Pn*	8.617*	5.524*	0.015
*Ci*	3.888	0.189	1.721
*Gs*	1.422	0.017	0.439
*Tr*	12.260**	0.239	0.071
Na-stem	8.005*	0.181	0.001
Na-leaf	4.976*	0.657	1.624
K-stem	0.079	1.855	0.046
K-leaf	0.557	0.039	0.557
Proline	6.048*	0.001	0.074

*P<0.05, **P<0.01, ***P<0.001.

Bold value means the trait was significantly affected by the treatment.

### 3.3 Physiological trait

**Figure 4:**
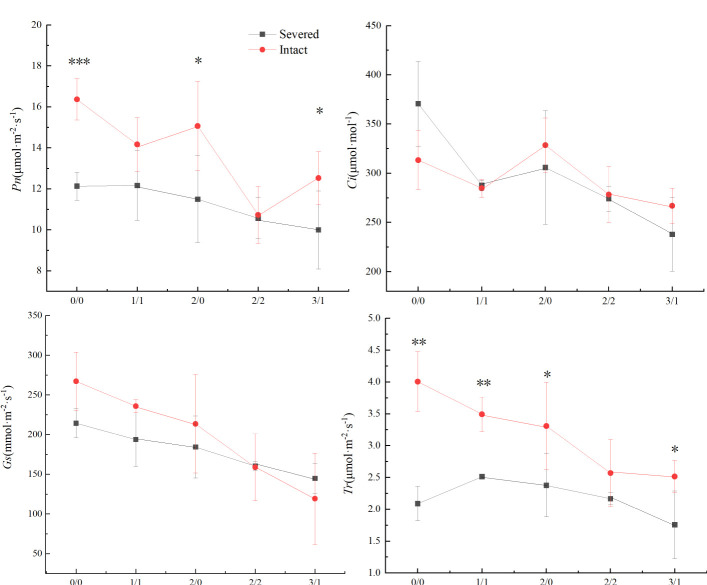
Effects of salt and severance on photosynthetic rate (*Pn*, a), intercellular CO_2_ concentration (*Ci*, b), stomatal conductance (*Gs*, c), and transpiration rate (*Tr*, d) of Phragmites australis under homogenous and heterogenenous salt treatments (mean ± SD). *, **, and *** represent significant differences between severed and intact treatments under the same salinity level at 0.05, 0.01, and 0.001 level.

*Pn*, *Gs*, and *Tr* were significantly affected by the salt and severed treatments (*p* < 0.05, **Figure 4**, **Table 1**). In the homogeneous environment, severed treatment significantly decreased *Pn* and *Tr* under the salt-free condition, and *Tr* in 1%/1% salt treatment (*p* < 0.01, **Figure 4**). Under heterogeneous salt conditions, severed treatment significantly decreased the *Pn* and *Tr* in both 2%/0% treatment and 3%/1% treatment (*p* < 0.05, **Figure 4**). In addition, heterogeneous salt treatment significantly affected *Pn* when compared with the homogeneous salt treatment (*p* < 0.05, **Table 3**).

**Figure 5:**
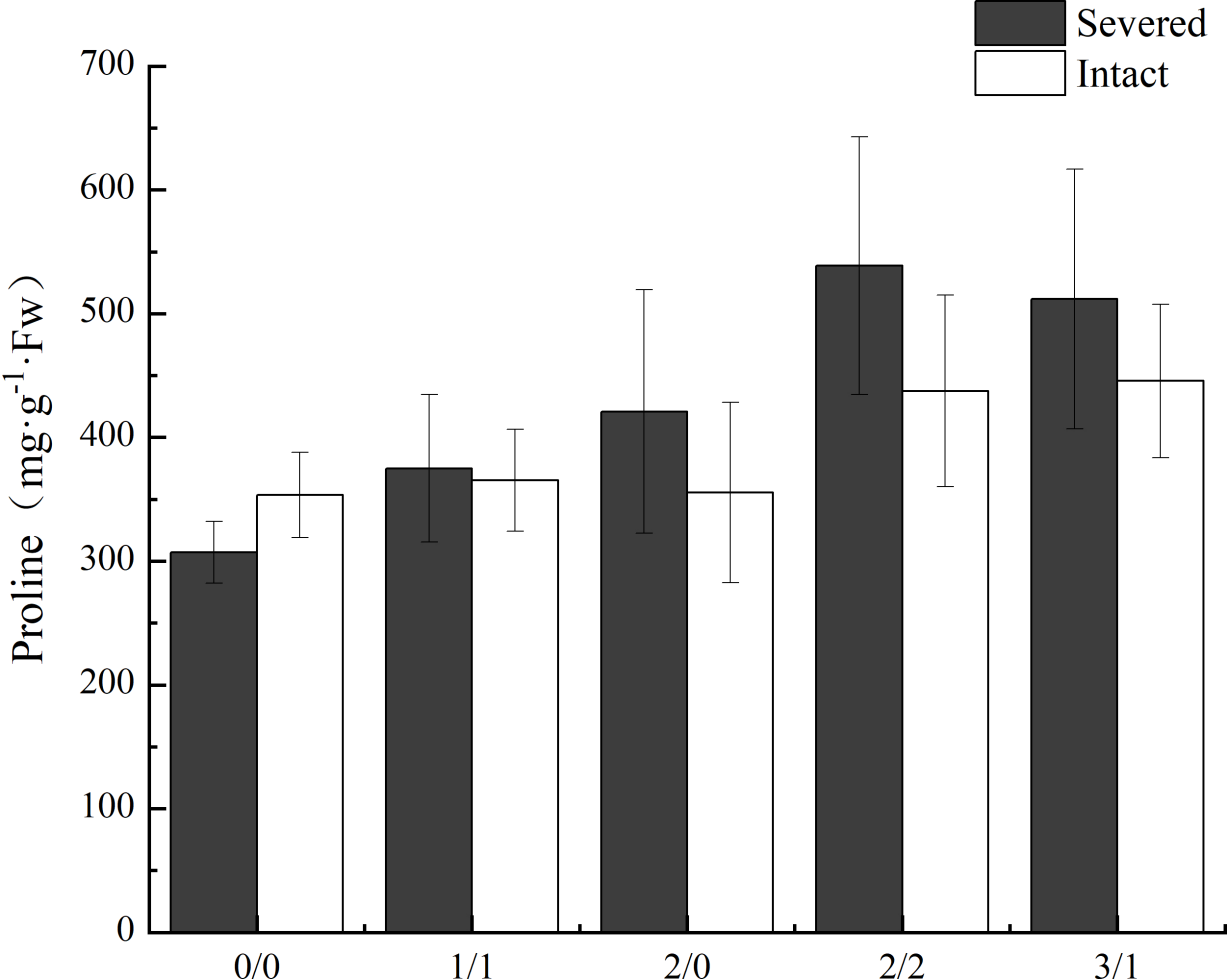
Effects of salt and severance on leaf proline content of *Phragmites australis* under homogenous and heterogenenous salt treatment (mean ± SD). Black (■) and white (□) column represent severed and intact treatment, respectively.

The proline content in leaves of *P. australis* was significantly increased by salt treatment (*p* < 0.05, **Figure 5**) in the homogeneous treatment, but was not affected by the severed treatment, even though the proline content was higher in the severed treatment (*p*>0.05, **Table 1**). No significant differences were observed between the homogeneous treatment and heterogeneous treatment (*p* > 0.05, **Figure 5**, **Table 3**).

**Figure 6:**
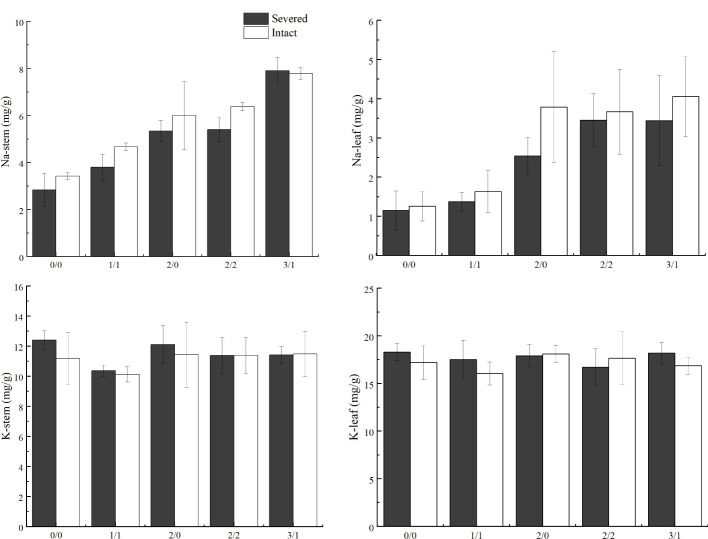
Effects of salt and severance on Na^+^ and K^+^ contents in stems and leaves of *Phragmites australis* under homogenous and heterogenenous salt treatments (mean ± SD). Black (■) and white (□) column represent severed and intact treatment, respectively.

Salt treatment significantly increased stem and leaf Na^+^ content in both the homogeneous and heterogeneous environments (*p* < 0.001, **Figure 6**, **Table 1**). Severed treatment significantly decreased stem and leaf Na^+^ contents (*p* < 0.01, **Figure 6**, **Table 2**). However, salt stress and severed treatment did not affect stem and leaf K^+^ contents of *P. australis* significantly (*p* > 0.05, **Figure 6**, **Table 1**, **2**), and a heterogeneous salt environment had no significant effects on the stem and leaf Na^+^ and K^+^ contents (**Table 3**).

## 4 Discussion

Among the environmental factors in coastal wetlands, salt is one of the major stress factors influencing plant growth and productivity (Li et al., 2018; Guan et al., 2021). In the present study, salt treatment significantly affected the growth of *P. australis*. With an increase in salt stress, plant performance decreased significantly, which has also been demonstrated by previous findings where salt stress significantly inhibited the number of stem nodes, plant height, leaf length, and leaf area in *P. australis* (Li et al., 2018; Ding et al., 2021; Sun et al., 2021), the growth potential of strawberry leaves (Saied et al., 2005; Turhan and Eris, 2009), and the tiller number, bud number, and rhizome length of *Leymus chinensis* (Zhang et al., 2015). However, in heterogeneous salinity environments, different types of plants have developed different salt adaptation mechanisms in the course of evolution; previous studies have demonstrated that clonal plants have a unique environmental adaptation mechanism through clonal integration (Chen et al., 2010; Xiao et al., 2010). Several studies have demonstrated that with the connections among ramets, clonal fragments can translocate resources and signals between ramets and increase the performance of the whole plant when exposed to a heterogeneous environment (Chen et al., 2010; Liu et al., 2017; Lu et al., 2020). In the present study, *P. australis* plant height in the severed treatment decreased significantly in the 0%/0% group and the 2%/0% heterogeneous salt treatment group. The relative density growth rate, plant height, leaf number, stem diameter, leaf length, and leaf width of *P. australis* exhibited a decreasing trend under the severed treatment, although the differences were not significant. Moreover, the aboveground biomass and underground biomass were affected significantly by severed treatment (**Table 1**, **2**). The results resolved our first question and demonstrated that clonal integration could alleviate the negative effects of salt stress on plants by increasing *P. australis* growth in saline environments. It could be explained that when clonal plants are in a stressful environment, they can balance stress factors (such as salt stress) and minimize the negative effects of the stress environment through clone integration (Beáta and Hubai, 2014; Wang et al., 2016; Waters et al., 2016).

Photosynthesis intensity is one of the key factors influencing plant metabolism, growth, and stress tolerance (Laplante et al., 2011). Environmental stresses (such as salt) could also affect plant photosynthesis by causing stomatal closure or damaging the photosynthetic structure and function (Meloni et al., 2003). In the present study, the *Pn*, *Ci*, *Gs*, and *Tr* of *P. australis* decreased significantly with an increase in salt stress. However, the *Pn* and *Tr* of *P. australis* were higher with than without ramet connection under the salt-free and heterogeneous salt treatments (2%/0% and 3%/1%). The results indicated that the photosynthesis parameters of *P. australis* were negatively affected by salt stress, but clonal integration could minimize the negative effects of heterogeneous salt stress (Xing et al., 2019). Conversely, higher photosynthetic capacities could support resource acquisition for plant growth and population expansion in heterogeneous environments (Wang et al., 2017; Xing et al., 2019).

In saline environments, compatible osmolytes, such as proline, have been shown to be fundamental in osmo-tolerance (Tabot and Adams, 2013; Guan et al., 2017). Such osmolytes, as well as other compounds, accumulate under environmental stress, such as salt stress or water stress, to alleviate the negative effects, and are found at high concentrations in plants adapted to dry or saline soils (Yukika et al., 1995; Santa-Cruz et al., 1999; Belkheiri and Mulas, 2013). Similarly, in the present study, salt treatment significantly increased leaf proline content, even under the heterogeneous salt treatment conditions. The results indicated that connected ramets may reduce salt stress through clonal integration. Xiao et al. (2011) also observed that leaf proline in the progeny after severing increased when compared with that in the progeny connected to the mother plant (Xiao et al., 2011). Other studies have also demonstrated that osmotic regulation is often coupled with reduced growth, which facilitates plant survival under salt stress (Belkheiri and Mulas, 2013; Guan et al., 2017). In the present study, the aboveground biomass in the severed treatment was lower than that in the intact group with the same heterogeneous salt treatment (such as the 2%/0% treatment); however, leaf proline content was higher in severed treatment.

Although stem and leaf Na^+^ concentrations in *P. australis* showed significant increases under salt stress in the present study, the modest alteration of Na^+^ concentration across a range of external Na^+^ concentrations indicated a superior capacity of *P. australis* to restrict Na^+^ uptake efficiently, which was also demonstrated by Pagter et al. (2009) and in our previous study (Guan et al., 2017). The capacity of plants to control salt concentration in tissues *via* reduced root uptake is a key mechanism that allows plant survival and growth under salt stress (Pagter et al., 2009). Notably, in the present study, clonal integration significantly increased stem and leaf Na^+^ accumulation in *P. australis* growing in the lower salt concentration patches, which were most likely imported from the connected ramets growing in the higher salt concentration patches. The results indicated that clonal integration could not only increase the concentration of nutrients, such as nitrogen, but also increase the contents of some ions, such as Na^+^, to a certain extent, and balance the salt ion concentration in the entire plant to adapt to the osmotic stress and ion toxicity, and, in turn, the external heterogeneous salt environment. Moreover, stem and leaf K^+^ concentrations showed no significant differences under each salt concentration gradient in the present study. The potential reason could be that the maintenance of K^+^ in aboveground tissue in the high Na^+^ environment is of great significance for the maintenance of the relative ion content balance, ensuring relatively normal photosynthesis and accumulating and transporting dry matter. Similar results have been reported in other species (Zhao et al., 2007; Silva et al., 2015; Sarker and Oba, 2020).

In conclusion, salt treatment affected *P. australis* growth significantly. However, salt heterogeneity did not have significant effects on morphological traits, but it decreased the aboveground and belowground biomass under 1% total salt concentration. Severed treatment intensified the adverse effects of salinity by decreasing photosynthetic capacity and biomass accumulation. According to the results, clonal plants can adjust their survival strategies in heterogeneous stress environments *via* morphological plasticity and clonal integration.

## Data availability statement

The raw data supporting the conclusions of this article will be made available by the authors, without undue reservation.

## Author contributions

BG, JBY and XZ designed the study. BG and MW conducted the control experiment. MW, XL and DZ carried out the data analysis and wrote the manuscript, and XW, JBY and JSY revised it. BG, XW and JBY coordinated the project. All authors contributed to the article and approved the submitted version.
